# Photoelectric Sensor for Fast and Low-Priced Determination of Bi- and Triphasic Segmented Slug Flow Parameters

**DOI:** 10.3390/s20236948

**Published:** 2020-12-04

**Authors:** Niclas von Vietinghoff, Waldemar Lungrin, Raphael Schulzke, Jonas Tilly, David W. Agar

**Affiliations:** Laboratory of Chemical Reaction Engineering, Department of Biochemical and Chemical Engineering, Technical University Dortmund, 44227 Dortmund, Germany; waldemar.lungrin@tu-dortmund.de (W.L.); raphael.schulzke@tu-dortmund.de (R.S.); jonas.tilly@tu-dortmund.de (J.T.); david.agar@tu-dortmund.de (D.W.A.)

**Keywords:** segmented slug flow, optoelectric sensor, online velocity measurement, multiphase systems, capillary reactor

## Abstract

Applying multiphase systems in microreactors leads to an intensification of heat and mass transport. Critical aspects of the well-studied segmented slug-flow, such as bubble generation and pump control, can be automated, provided a robust sensor for the reliable determination of velocity, phase lengths, and phase ratio(s) is available. In this work, a fast and low-priced sensor is presented, based on two optical transmission sensors detecting flow characteristics noninvasively together with a microcontroller. The resulting signal is mainly due to refraction of the bubble-specific geometries as shown by a simulation of light paths. The high performance of the processing procedure, utilizing the derivative of the signal, is demonstrated for a bi- and triphasic slug flow. The error of <5% is entirely reasonable for the purpose envisaged. The sensor presented is very fast, robust, and inexpensive, thus enhancing the attractiveness of parallelized capillary reactors for industrial applications.

## 1. Introduction

Multiphase systems in chemical processes, such as extractions, absorptions, and reactions, often suffer from stochastic surface areas and/or low mass and heat transport rates, which limit their efficiency [1]. Microcapillary reactors with segmented slug flow exhibit excellent behavior in such multiphase systems. Segmented slug flow has been extensively studied in circular capillaries (≤1 mm inner diameter) [2,3,4,5,6,7,8]. Each phase involved forms a regular, stable segment within a uniform alternating sequence, where a bubble is defined as a droplet and a slug as the continuous phase between two bubbles, both with an axial length greater than its diameter. Due to its regular and specific form, the interfacial surface area and the surface-to-volume-ratio are both large and well defined. In addition, the small dimensions and internal circulations intensify the transport phenomena. Segmented slug flow can be generated for biphasic liquid/liquid and liquid/gas systems or even triphasic liquid/liquid/gas systems [4]. To summarize, the segmented slug flow in capillary reactors seems to offer a promising alternative to conventional multiphase unit operations [9].

However, the capacity of the capillary reactor remains a challenge. With flows of just a few milliliters per minute in a single capillary, the throughput is inadequate for most production-scale plants. To increase the capacity, a scale-up by increasing dimensions is not feasible, since the advantages of this reactor concept would no longer pertain. The alternative strategy adopted is numbering up, a parallelization of single capillary reactors together with distribution units for each phase. Distribution units are preferred to decrease the number of pumps necessary and, thus, the costs. For single-phase systems, the distributor design has already been optimized so as to minimize the maldistribution of the volumetric flow rate and, thus, harmonize the conditions inside each capillary [10,11,12]. By just modifying the design, the systematic maldistribution of the volumetric flow rate for more than 100 parallelized channels can be reduced to around 5% [10]. Multiphase systems exacerbate this nonuniformity. As a function of the maldistribution for the single-phase systems, different slug/bubble lengths and phase ratios arise in individual channels, leading to different pressure drops due to the extent of the phase interfaces in each channel, which further increases the maldistribution. Even by changing the design of the distributor for the two-phase flow by increasing the pressure drop within the manifold, a maldistribution could not fully eliminated [13]. Nevertheless, it has been demonstrated that the inhomogeneities of biphasic liquid/liquid flow in numbering up can be suppressed by feedback control of the volumetric flow rate and slug/bubble lengths in each individual channel using a temperature-regulated pressure drop element and a step-motor-driven coaxial slug generator [3]. This technique necessitates actuators and sensors for each channel in the numbered up system.

Although it has been demonstrated that a numbering up of capillary reactors on the basis of distribution units is feasible, the segmented slug flows in capillaries have to provide an affordable option for multiphase systems in the chemical industry. Thus, low-priced and robust actuators and sensors are essential if capillary reactors are to be an attractive practical solution. Invasive sensor systems, such as a hot-wire anemometer, are fundamentally unsuitable, since they can affect and disrupt the advantageous flow pattern sought. Further work, thus, needs to be focused on noninvasive sensors for monitoring velocities, phase ratios, and slug/bubble lengths.

**Figure 1:**
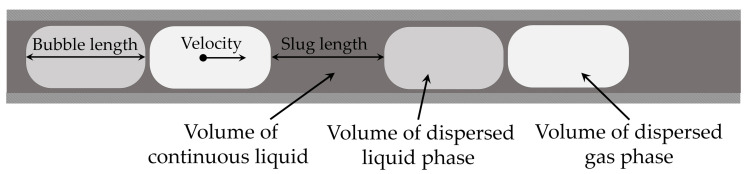
Schematic of a triphasic slug flow with two liquid components and one gaseous component. The phase ratios can be determined either by a ratio of the slug and bubble lengths or, more appropriately, by the ratio of the phase volumes. The volumetric flow rate can be calculated using the velocity and the cross-sectional area together with the wall-film thickness. The slug/bubble lengths and velocity are, thus, required to control volumetric flow rate and phase ratio. The velocity is defined as mean velocity from the cross-sectional area and assumed to be constant for all phases within a fully developed slug flow.

To begin with, Figure 1 depicts the variables to be controlled in slug flow. It should be noted that the dispersed phase bubbles are enclosed by the better wall-wetting continuous phase, which usually forms a thin wall film. In this work, the continuous phase is also considered as a slug, the length of which can be measured. The wall film must nevertheless be taken into account if a volumetric flow rate is to be determined from the velocity. For this purpose, existing models for the thickness of the wall film can be applied to calculate the volumetric flow rate, which is useful, for example, if a pump is to be controlled [14,15,16].

Multiple strategies have already been implemented to determine characteristic parameters from the segmented slug-flow. Tracking the slug flow with a high-speed camera is one possibility. High resolution of both the image and the time, together with adequate image processing, yields slug/bubble lengths, phase ratios, velocity, and bubble shape. The raw image data are usually converted to a binary grayscale facsimile, which permits the various phases to be distinguished. The number of pixels enables one to deduce the slug and bubble lengths. Together with the subsequent snapshot and the frame rate, the velocity may then be calculated [17,18]. This method determines precise values for the acquired data. However, since high-resolution images and high frame rates are needed, the costly camera required is an inappropriate sensor system for an industrial numbering up task. Flow characterization by microparticle image velocimetry (µPIV) could also be used to ascertain the data required and would enable visualization of internal slug flow patterns [19]. This technique is, however, similarly too expensive for the purposes intended. Both camera tracking and µPIV utilize time-dependent two-dimensional (2D) images, thus increasing the complexity of data processing. These techniques are primarily suitable for obtaining detailed insights into the flow in laboratory-scale equipment and for calibrating other sensors. On the other hand, time-dependent zero-dimensional (0D) sensor systems offer a viable alternative. These 0D sensors monitor only a single location along the capillary, which all the phases of interest flow past. Two major 0D tracking systems have been investigated for segmented slug flows, according to variations in either electrical (conductivity) or optical behavior (i.e., transmissive sensors: absorption, refraction) for each phase involved.

**Figure 2:**
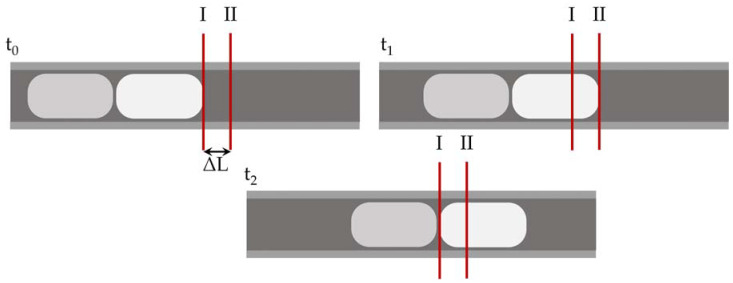
Principle of a zero-dimensional (0D) segment length and velocity sensor. In this case, the length and velocity of the first white bubble is measured. At t_0_, the front cap of the bubble passes by the first sensor I. At t_1_, the same feature passes sensor II. At time t_2_, the rear cap of the bubble passes the sensor I. With the help of the time differences, the velocity and bubble length of this bubble can be calculated using the equations given.

The measuring principle of 0D sensors is illustrated in Figure 2. The sensors must be able to distinguish between the passing phases. For velocity and length measurements, two sensors on one capillary are required. The quotient of the separation between the sensors ΔL and the time difference t_Δ_ = t_1_ − t_0_, which a specified characteristic point of the flow (e.g., a bubble cap) requires to flow from the first sensor to the second, yields the slug velocity as

(1) $$u_{SL}=\frac{∆L}{t_{∆}}.$$

Using the slug velocity u_SL_, the slug or bubble length l_i_ can also be calculated. This is obtained by multiplying the time t_Pi_ = t_2_ − t_0_ required by one segment being considered to pass one of the sensors.

(2) $$l_{i}=u_{SL}\times t_{Pi}.$$

With this method, the length of each phase can be determined. Using the ratios of the lengths for the individual phases, the phase ratios can be evaluated.

Conductivity sensors, such as the commercially available capacitively coupled contactless conductivity detection (C^4^D) sensor, employ a contactless measurement of the medium conductivity in a capillary with two external electrodes [20,21,22,23]. This measurement method is particularly elegant, since it can also be utilized for nontransparent capillaries and is well suited for two-phase systems [22,23]. A disadvantage, however, is that at least one of the phases (usually an aqueous phase) must have good conductivity [20,22]. A liquid/gas slug flow with a nonconducting oil as the liquid phase, for example, cannot be measured using this method. In a three-phase liquid/liquid/gas flow, which normally only has one conductive phase, only information on the conductive phase can be captured. Measurement via contactless conductivity sensors is, therefore, suitable for some two-phase systems, but not generally applicable.

Photoelectric sensors, comprising one light emitter and one photodetector, represent an even simpler measurement technique. The emitted light is transmitted through the capillary and the flow to be evaluated. As a consequence of substance-specific light refraction, reflection, or absorption, different light intensities are detected. On the basis of the resultant temporal change of the light intensity, individual phases can be clearly assigned to certain detection levels. By using two photoelectric sensors, the flow velocity and slug/bubble lengths can be calculated for a biphasic segmented slug flow [3,24,25]. Mithran and Venkatesan configured one transmissive sensor horizontally and another vertically to acquire additional information about the wall-film thickness [24]. Harvie and de Mello ascribed the main influence on the signals to light refraction and reflection, which in turn depended on the substance-specific refractive indices and the geometry of the phase interfaces [25]. The phases can, thus, be detected independently of their conductivity and, in theory, the differentiation between three phases is, therefore, also possible. Harvie and de Mello employed centered positions for the detectors when setting up their sensor. Changes in this position could lead to an optimization of the resulting signal in terms of light refraction, because, in a centered location behind the capillary, the light beams are focused, which renders a differentiation of the phases more difficult, depending on the substances comprising the system. A smaller photodetector can also lead to a higher sensitivity of the capillary position with respect to the photodetector. Commercial “forked” photoelectric sensors (i.e., TCUT1300X01, Visahy) are particularly appealing, because they are photodetectors having an edge length of 0.3 mm and a distance between emitter and detector of 3 mm, which is well suited for microcapillaries [26].

The approach of using double transmissive sensors as a sensor system for slug flows appears to be one of the most effective and economical ways to detect slug-flow characteristics. In light of recent research on three-phase slug flows, the sensor system is extended from two-phase to encompass three-phase flows in this work [2,4,27]. In this context, the influence of capillary position relative to the photodetector is also examined, since an improvement in the discrimination between the individual phases is expected as a function of the light refraction. A forked photoelectric sensor is used for this purpose. Since two transmissive sensors are required for the determination of the velocity and the slug length, the degree of freedom with respect to the distance between the transmissive sensors is also discussed. In order to compensate for small variations in the signal level (e.g., due to a change in the refractive index as a result of reaction), a data evaluation method is presented, which operates independently of the signal level and automates the acquisition of the required parameters.

## 2. Materials and Methods

Since the slug flow represents the underlying flow pattern to be examined, the flow generation is first described; then, the components of the sensor are detailed before, lastly, the assumptions and approaches for the calculation of the beam path through the capillary are elucidated.

The triphasic flow comprises the three immiscible fluids: water (double-distilled), 1-hexanol (99% purity, Alfa Aesar, Kandel, Germany), and nitrogen (99.999% purity, Messer, Bad Soden, Germany). The two liquid phases are delivered with 50 mL syringes (Syringe Gastight 50mL, SGE Analytical Science, Ringwood, Australia), by means of a syringe pump (Legato 100, kd Scientific). A mass flow controller (F-201-CV, Bronkhorst, Ruurlo, Netherlands) is used to adjust the volumetric nitrogen flow. Transparent fluorinated ethylene propylene (FEP) capillaries (1 mm inner diameter (ID), 1/16″ outer diameter (OD)) are used by virtue of their excellent hydrophobic and optical properties and because of their outstanding chemical resistance.

**Figure 3:**
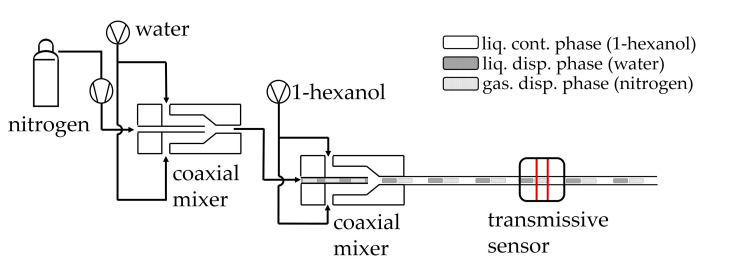
Schematic of the set-up used to generate the triphasic segmented slug flow. Left: Generating a biphasic slug flow of water and nitrogen in the first coaxial mixer. Middle: The continuous phase 1-hexanol encircles in the second coaxial mixer the biphasic flow generating a triphasic slug flow. Right: Detection of the slug flow with a transmissive sensor.

To generate a stable and uniform triphasic flow, as described by Hellmann et al., two coaxial slug generators are placed in series [4]. By varying the position of the inner capillary in these coaxial slug generators, the bubble lengths can be adjusted reproducibly, flexibly, and independently of the volumetric flow rates and phase ratios [28]. The optical sensor is placed about 30 cm downstream of the second slug generator, thus ensuring stable fully developed slug flow, where all segments exhibit the same cross-sectional velocity. A high-speed camera (DMK 23G618, Imaging Source, Charlotte, NC, USA) is installed just behind the optical sensor, which is used for the subsequent validation of the data measured with the optical sensor. In Figure 3, the experimental set-up is presented schematically.

The optical sensor used, a transmissive optical sensor equipped with two channels (TCUT1300X01, Vishay, Malvern, PA, USA), has a forked arrangement incorporating one emitter (emitted wavelength: 940 nm) and two detectors (phototransistors). Detailed dimensions and specifications can be found in its technical specifications [26]. This optical sensor was firmly soldered onto a circuit board.

**Figure 4:**
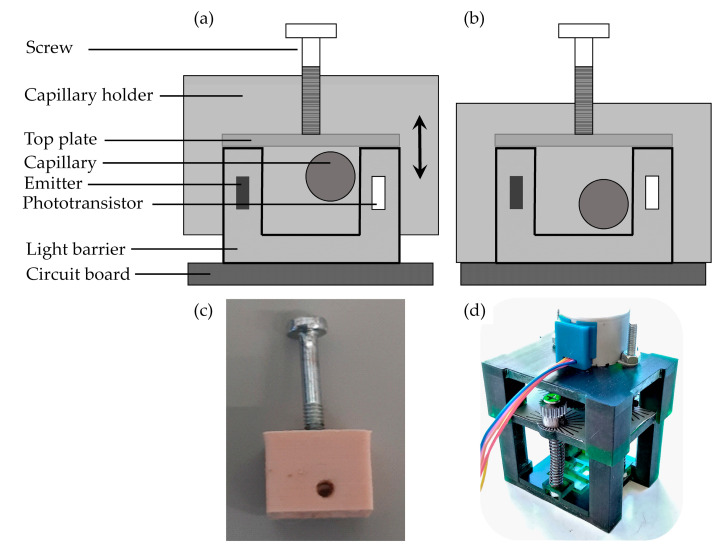
Schematic drawing and photos of the capillary holder. By pressing the screw against the stationary top plate, the holder (including capillary) can be lowered and raised relative to the transmissive sensor. (**a**) Capillary in highest position; (**b**) capillary in lowest position; (**c**) three-dimensional (3D) printed capillary holder; (**d**) capillary holder combined with step motor.

In order to be able to adjust the exact position of the capillary evenly and in small steps, a capillary holder was fabricated, which allows small reproducible changes in the height of the capillary. In Figure 4a,b, the capillary holder is presented schematically, and, in Figure 4c, a photo of the three-dimensional (3D) printed holder made of polylactic acid (PLA) is shown. A recess is provided inside the capillary holder, the dimensions of which are selected so that the transmissive sensor unit can be completely embedded in the capillary holder. At the lowest position, it rests on the circuit board. A small top plate is attached on top of the transmissive sensor unit, which prevents the adjusting screw from protruding between the emitter and detector. In addition, this plate distributes the load evenly between the emitter and detector housings. The capillary holder contains a hole through which the capillary is passed and, thus, fixed in space. This hole is placed in such a way that, when the screw is completely unscrewed, the capillary is located at the bottom of the transmissive sensor. The decision with respect to the horizontal positioning of the capillary close to the transmitter is based on the theoretical consideration that the aperture in front of the detectors has the greatest effect when the capillary is positioned directly in front of the aperture. For detailed analysis of capillary position, a capillary holder together with a step motor (Figure 4d) adjusting the screws was constructed, which allows an automated and uniform positioning of the capillary.

The functional principle of the prototype constructed in this way is that, by screwing in the adjusting screw, the construction—including the fixed capillary—is pushed up evenly via the plate on the transmissive sensor housing. The screw is connected to the holder with a nut, which is attached to it. In addition, the capillary holder shields the transmissive sensor from the extraneous influences of external light.

With this capillary holder, the vertical position can be adjusted by a screw with a pitch of 0.5 mm, thus varying the height of the capillary by 0.125 mm per quarter rotation. Hence, the influence of the vertical capillary position on the signal quality can be investigated systematically.

The optical sensor is connected to a microcontroller board (teensy 4.1, PJRC, Sherwood, OR, USA), where the signals of the transmissive sensors are digitalized with a 12 bit resolution. The data evaluation to determine the velocity and lengths is also performed on the microcontroller board (see Section 3.2). The resulting data can either be stored on a micro secure digital (SD) card or transferred serially to a personal computer.

MatLab 2020a was used to simulate the light beam paths (see Section 3.1). Firstly, the angle of incidence was determined at which a light beam impinges upon a phase interface. For simplification, it was assumed that, depending on the angle of incidence and the refractive indices of the substances involved, the light beam is either completely refracted or completely reflected.

Of the materials used (FEP, 1-hexanol, water, nitrogen, and air), nitrogen and air show the greatest difference to the others in their refractive indices (see Table 1). Based on this and Fresnel’s formulae, total reflection is only assumed if the incoming light beam from an optically denser medium (1-hexanol, water, or FEP) passes into one of the optically less dense phases (nitrogen or air) [29]. Total internal reflection occurs only if the angle of incidence exceeds a critical value. The critical angle α_c_ depends on the ratio of the refractive indices and is determined by the following relationship (where *n*_2_ < *n*_1_ applies):

(3) $$\alpha_{c}=\;arcsin\left(\frac{n_{2}}{n_{1}}\right).$$

If the ratio of the refractive indices is greater than 1, the proportion of reflected light is low according to Fresnel and only becomes relevant at angles close to 90°. For this reason, only the refraction of light is taken into account, when light passes from an optically less dense medium (nitrogen or air) to an optically denser one (1-hexanol, water, or FEP).

If the light is refracted, Snell’s law of refraction is used to calculate the exit angle [32].

(4) $$\frac{sin\left(\alpha_{i}\right)}{sin\left(\alpha_{j}\right)}=\frac{n_{j}}{n_{i}}.$$

Parallel light rays impinging upon the capillary are considered. A parallel light beam alignment can be assumed, since the light source is relatively far away from the capillary and no other light sources are present. The following dimensions of the capillary are assumed: 1/16″ OD, 1 mm ID, FEP. In the capillary, either a pure 1-hexanol phase or a discrete bubble (nitrogen or water), which is separated from the inner wall of the capillary by a wall-film of 1-hexanol is observed. The thickness of the wall film is determined with the help of the model proposed by of Aussillous and Quéré to be 90 µm [16]. The whole system of capillary, wall film, and bubble is assumed to exhibit rotational symmetry.

## 3. Results

In order to for the optical 0D sensor to be considered a feasible cost-effective alternative to an industrial multiphase capillary reactor system, it is first investigated how the raw signal from the sensor can be reproducibly adjusted and improved. The resulting data are then interpreted by simulated beam paths through the capillary. Next the data processing is explained, and a robust and rapid technique for this step is presented. Subsequently, the distance between the two sensors required is discussed, which has a direct influence on the velocity calculation. Lastly, the velocities and length determined by the sensor developed are evaluated according to their accuracy.

### 3.1. Influence of Vertical Capillary Position

Since the optical detector used (detector: 0.3 mm width 0.3 mm height; aperture: 0.3 mm width, 1 mm height) is considerably smaller than the capillary diameter (1/16″), a detailed analysis of its position with respect to the capillary in relation to the form of the signal is possible. This degree of freedom allows one to manipulate the signal so as to optimize the data processing for more accurate measurements.

**Figure 5:**
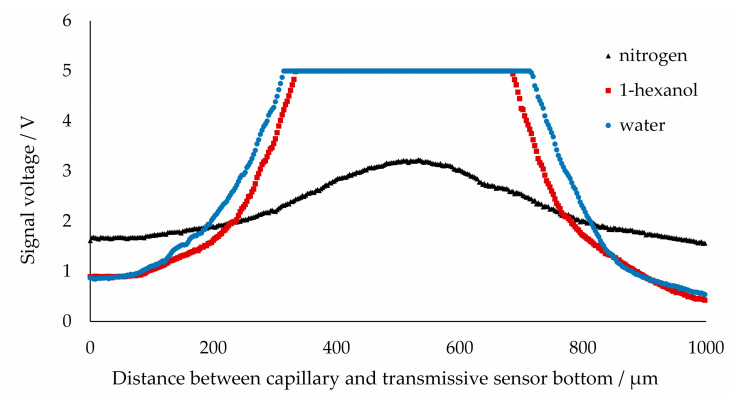
Measured voltage from phototransistor for a single-phase 1-hexanol-filled capillary and water or nitrogen bubble with uniform capillary vertical displacement using a step motor.

First, the height of a capillary containing a single phase is adjusted and the resulting voltage observed. For this purpose, a segment of the phase of interest was positioned in the transmissive sensor by stopping the pumps. The capillary was then shifted evenly from the lowermost to the uppermost position by using the capillary holder with a stepper motor. At the lowest position, the capillary lies on the bottom of the transmissive sensor. Figure 5 shows the resulting voltage of the transmissive sensor at different heights. If the capillary is centered (~550 µm) in front of the detector, the signals for the liquid phases are 5 V and, thus, at the detection limit and significantly higher than the baseline signal (emitter to detector without capillary) of 4 V. If the capillary is displaced vertically away from the detector, the resulting signal drops sharply. Proceeding from the centered position of the capillary, this drop is roughly symmetrical with respect to the upward and downward displacement. Nitrogen also exhibits a maximum signal for the centered capillary but does not attain the upper detection limit.

This observation can be explained by purely optical considerations. It is assumed that the refraction of light is the decisive issue dictating the signal. Thus, the signal is only dependent on the system geometry, the wavelength of the light, and the refractive indices of the media.

**Figure 6:**
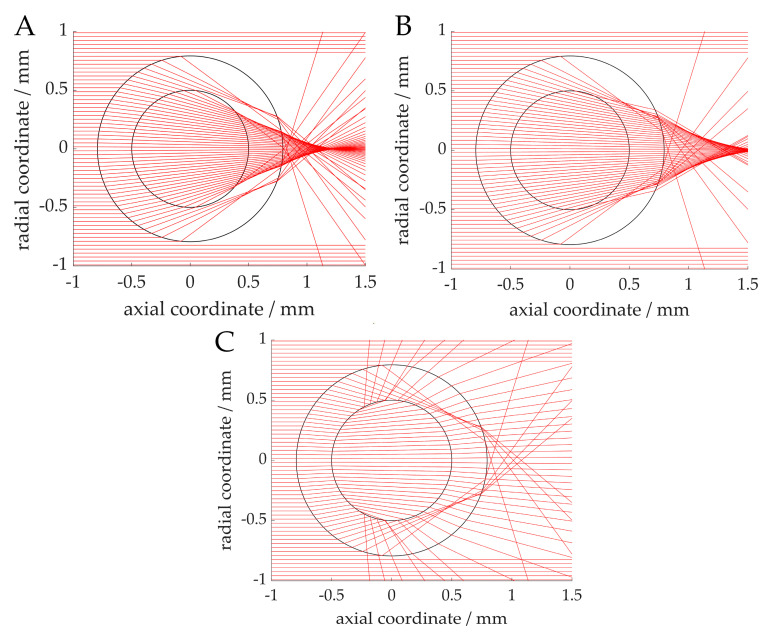
Simulated light refraction through capillary in vertical cross-sectional view. External medium: air, capillary: FEP, wall-film: 1-hexanol (not visible due to its low thickness), internal medium: 1-hexanol (no wall film, (**A**)), water (**B**), nitrogen (**C**). The phototransistor can be assumed to be located at approximatively the 1 mm axial coordinate.

Simulations of the light beams through the capillary lead to light refraction, as shown in Figure 6. The simulated beam paths through 1-hexanol and water (Figure 6A,B) show a focusing of the light in the center of the capillary. This explains why the recorded voltage (see Figure 5) is higher than the baseline signal of the transmissive sensor without the capillary. The sharp drop at about 300 and 750 µm can also be explained by this argument, because the rays are deflected away in these regions. The drop can be seen to occur at an earlier stage in the case of 1-hexanol, which can be attributed to a stronger focusing. In the gas phase (Figure 6C), no light focusing but a slight divergence can be identified. In addition, some light rays are reflected from the phase transition of the wall film (1-hexanol) to nitrogen due to the large angle of incidence. This explains the low signals compared to the liquid phases. A higher signal in the center of the capillary results from the light focusing of the rays, which only pass through the capillary (FEP) and do not enter the gas phase.

**Figure 7:**
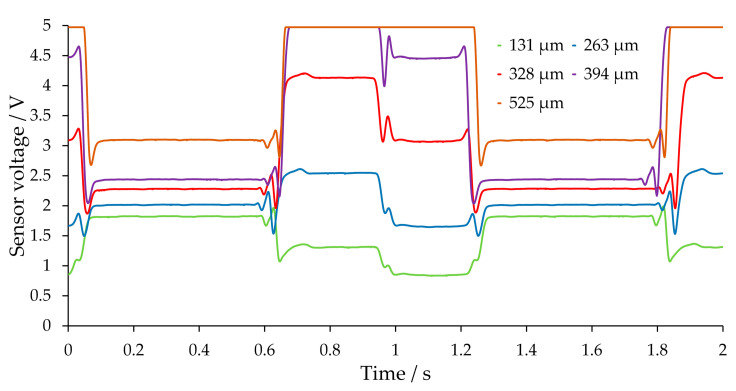
Measured voltages at different positions of the capillary relative to the phototransistor. The height is the distance between the lowest capillary position in the transmissive sensor and its actual position. Shown are positions from the bottom to an approximately centered position at 525 µm. Plateaus: nitrogen (0.1–0.6 s and 1.3–1.8 s), water (0.6–1.0 s and from 1.8 s), 1-hexanol (up to 0.1 s and 1.0–1.3 s). The lengths of the plateaus show slight deviations due to deviations in bubble and slug length. The raw signal was processed with a moving average filter (see Section 3.2).

Since a large influence of the position of the capillary vis-à-vis the detector was already observed in a resting slug flow, dynamic signals for different capillary positions were also recorded for a triphasic flow. In Figure 7, the signals for a triphasic flow are shown. The gas phase was chosen to be particularly long in order to be able to assign it unambiguously to a particular voltage plateau. Furthermore, it was observed that the gas phase is followed first by a water bubble and then the continuous phase of 1-hexanol, which also facilitates the identification of the liquid phases from the signal.

The effect of the light being focused can be observed in the case of the centered position of the capillary and, thus, the amplitude of the resulting signal (see Figure 7, capillary position at 525 µm). This effect is nevertheless unsuitable for an evaluation, since it is no longer possible to discriminate between the two liquid phases. An exception is the nitrogen bubble, which has a low signal at most heights and, thus, a low transmission. If the deflections in the vertical cross-section of the capillary with a nitrogen bubble are considered (Figure 6C), the divergence and reflections lead to no focusing and, thus, to a low voltage. Since this effect depends only partially on the capillary position, the signal for the gas phase is consistently at a low level.

If the capillary moves away from the centered position (~550 µm centered position), all three phases may be distinguished. With such a signal, it is also possible to distinguish between the individual phases very clearly. Each phase present can be assigned to a given signal plateau. The signal at position (328 µm) is particularly suitable, due to the large differences in amplitude.

**Figure 8:**
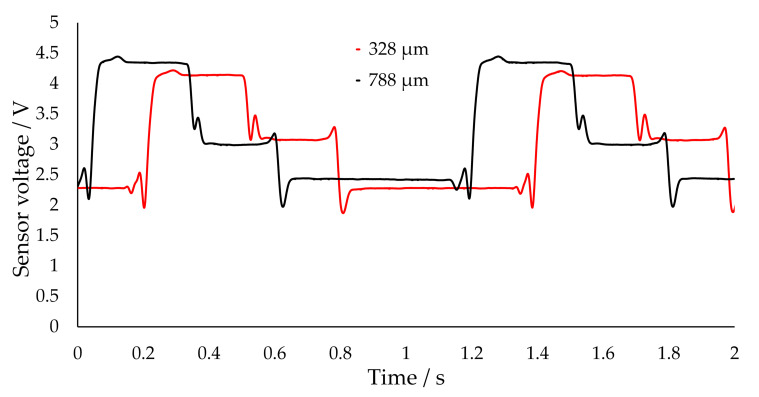
Measured voltages for two positions of the capillary relative to the phototransistor. The height is the distance between the lowest capillary position in the transmissive sensor and its actual position. Shown are positions with approximatively the same distance to the centered position. Plateaus: nitrogen (~2.3–2.5 V), 1-hexanol (~3.0–3.2 V), water (~4.2–4.4 V). The signals where shifted with respect to time for a better comparison. The raw signal was processed with a moving average filter (see Section 3.2).

Furthermore, if the capillary position is moved from the centered position in the other direction, a symmetry of the signals can be observed in Figure 8, such that the signal depends only on the distance from the centered position and not on the direction of the displacement (up or down). The signals at 328 µm and 788 µm are almost identical due to a similar distance to the centered position.

**Figure 9:**
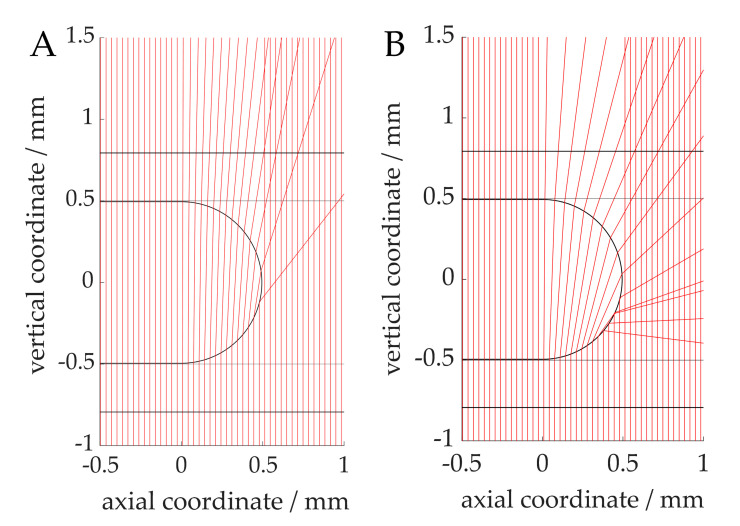
Simulated light refraction through capillary in horizontal cross-sectional view. External medium: air, capillary: FEP, wall film: 1-hexanol (not visible due to its low thickness), continuous medium: 1-hexanol, bubbles: water (**A**), nitrogen (**B**). The phototransistor can be assumed to be located at approximatively the 1 mm radial coordinate.

At the respective ends of the phase plateaus, minima and maxima are also visible in the signal. This signal shape can also be explained by the light refraction. Figure 9 shows the beam path in the horizontal cross-section of a capillary with bubbles. Although only hemispheres were assumed as bubble caps here, which does not completely correspond to reality, the effects of light refraction are nevertheless well represented [33]. At the cap of the water bubble (Figure 9A), a slight deflection of the rays can be observed. The deflection at the level of the bubble cap results in a minimum in the signal, since, at this point, less light transmission can be measured than before and after the bubble. In this case, the light focusing further to the right of the cap explains the maxima of the signals. In the case of the nitrogen bubble (Figure 9B), the deflection of light is stronger due to the greater difference in refraction, and the effect is correspondingly greater. These phenomena and the alignment of the individual segments result in the transition zones between the phase plateaus. In this case, the exact shape depends on the substance-specific refractive indices and the geometric shapes. The shape of the bubble cap may differ depending on the constituent substances and the flow velocities.

The distinct plateau heights result from the different light beam deflections, which can also be seen in the vertical cross-sections. Since an absorption by the substances involved can be considered as low (see ultraviolet–visible light (UV–Vis) spectra, Appendix A), the resulting signals are predominated on the basis of the different refractive indices, the geometry, and the capillary position with respect to the detector.

With the capillary holder and the positioning relative to the detector, the signal can be adjusted so that all phases can be clearly distinguished from one another. Using these signals, the data evaluation described in Section 3.2 can now be used to determine the flow velocity, slug/bubble lengths, and phase ratios.

### 3.2. Mathematical Data Processing

**Figure 10:**
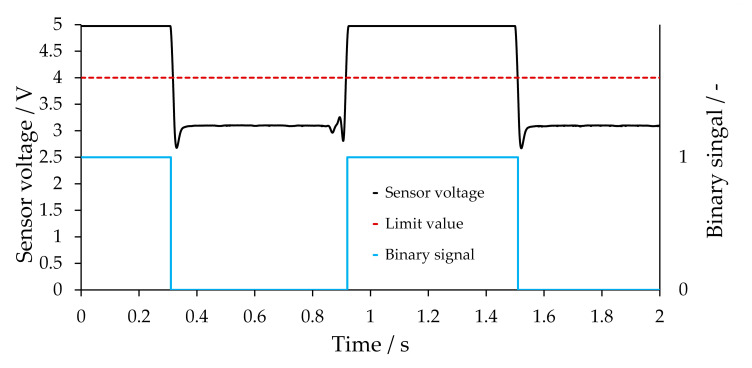
Transmissive sensor voltage (filtered with moving average) and its corresponding binary signal at a position of 525 µm (see Section 3.1). The high plateau can be assigned to the liquid phases, while the low plateau can be assigned to nitrogen. The limit value was chosen to be 4 V.

Having established good fine-tuning of the signal via the capillary height, the improved signal can now be used to calculate the slug velocity. The simplest method initially used is to set limits between the signal plateaus, which can be related to the respective phases. If the limit values are exceeded or not attained, the signal section is assigned to a particular phase. Thus, the signal of a biphasic flow can be transformed into a binary square-wave signal, and the signal of the triphasic flow can be correspondingly transformed into a ternary square-wave signal. With the square-wave signals, the flow velocity and slug/bubble lengths can be determined from the distance between two transmissive sensors. If this method is used, the signal height of the respective phase plateaus is required to define the limit values. This is illustrated in Figure 10 using the signal at the centered position (Figure 7, 525 µm), which presents the differentiation between gas and liquid, as in a biphasic flow. The limit value can, for instance, be the average value of the two-phase plateaus. A high measuring frequency together with measurement noise can lead to a limit value being exceeded several times within a short period. A filter is, therefore, used to smooth out the measured signal. In this particular case, a moving average filter was used. The number of averaged values is set depending on the measuring frequency. At a measuring frequency of 9600 Hz, for example, the last 128 values (corresponding approximately to the values of the last 13.3 ms) are averaged.

However, this technique demands that the plateau heights remain constant. As soon as these change, with other media for instance, a calibration to establish the new plateau heights is necessary. As shown above, small differences in height of the capillary relative to the detector can have a large effect on the signal amplitude. Since it was shown that the refractive index also has a significant influence on the signal, a change in the refractive index may also necessitate a fresh calibration. This is especially awkward if the refractive index changes axially within the capillary, due to reaction, extraction, or temperature variation. Often, these factors fluctuate dynamically and can change according to the exact mode of operation. New limits must also be defined for each new system of media. Thus, the evaluation of the signal may be suitable in some cases, but it is not flexible enough to be generally applicable. In order to eliminate this lack of robustness, an alternative evaluation method is now presented, which can account for variations in the plateau height without recalibration.

**Figure 11:**
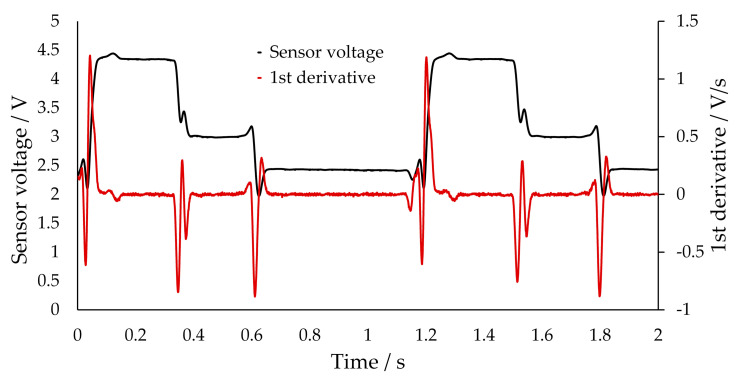
Transmissive sensor voltage (filtered with moving average) and its first derivative for a triphasic segmented slug flow at a position of 788 µm (see Section 3.1). The plateaus can be assigned to the phases as follows: water, 1-hexanol, nitrogen in the direction shown from left to right; the nitrogen plateau is the longest.

If the first time derivative of the smoothed signal is considered, the phase plateaus are located approximately on the *x*-axis. However, the flanks represent a clear boundary between the plateaus. The flank describes the transition between the two phase plateaus, which is normally characterized by a steep gradient. The derivative is, therefore, independent of the plateau height, and the phase differentiation can be performed exclusively on the basis of the flanks. A calibration based on the plateau height is, therefore, no longer necessary. For the calculation of the slug velocity, the time t_Δ_ is needed, which the phase under consideration needs to pass from the first to the second transmissive sensor. Therefore, a characteristic point in the recorded signal must be identified. During the latter validation (see Section 3.4), it is shown that, with the measurement accuracy for the velocity, it is sufficient to use the transition between the plateau and the following flank. Therefore, a distinction must only be drawn between plateau and flank in the data. Figure 11 depicts the derivative of a signal for a three-phase flow.

For the slug or bubble length, the exact time of the beginning and end of the cap is needed. Using the results from the simulated light paths through the capillary, it can be assumed that a bubble cap creates a minimum in the signal due to the divergence of the light beams (see Section 3.1, Figure 9). Indeed, one minimum can be observed in the signal between a bubble and the continuous slug. At the transition between nitrogen and water, two minima can be observed, because a thin layer of continuous phase (1-hexanol) separates both phases; thus, two bubble caps (for water and nitrogen) arise at this point. To obtain the time t_Pi_, which is needed for the phase length calculation (Equation (2)), the time between the two minima surrounding a plateau is utilized. The minima can be derived from the first derivative. However, the derived slug or bubble length has to evaluated for a new substance system since the cap shape may change. In this case, other characteristic points in the signal may also be used to obtain an appropriate slug/bubble length value.

Slug and bubble lengths and velocities can, thus, be unequivocally determined. However, the precondition for the clear identification of the phases involved remains. In a three-phase flow, therefore, three phase plateaus must still be identifiable. For this purpose, the direction of the slope can be used. If the average slope direction (positive, negative) is determined for the flanks, only two sequences of slope direction of the flanks are possible: either positive(I), positive (II) and negative (I) or negative (I), negative (II) and positive (I). Through an initial observation, the phases can be assigned to the transitions of the slope directions of the previous flank. For example, in Figure 11, the assignment is as follows: nitrogen (negative (II) → positive), water (positive → negative (I)), and 1-hexanol (negative (I) → negative (II)). As long as the segment sequence along the capillary does not change, no further calibration is necessary. The data evaluation is, therefore, more robust than the calculation via the limiting values.

**Figure 12:**
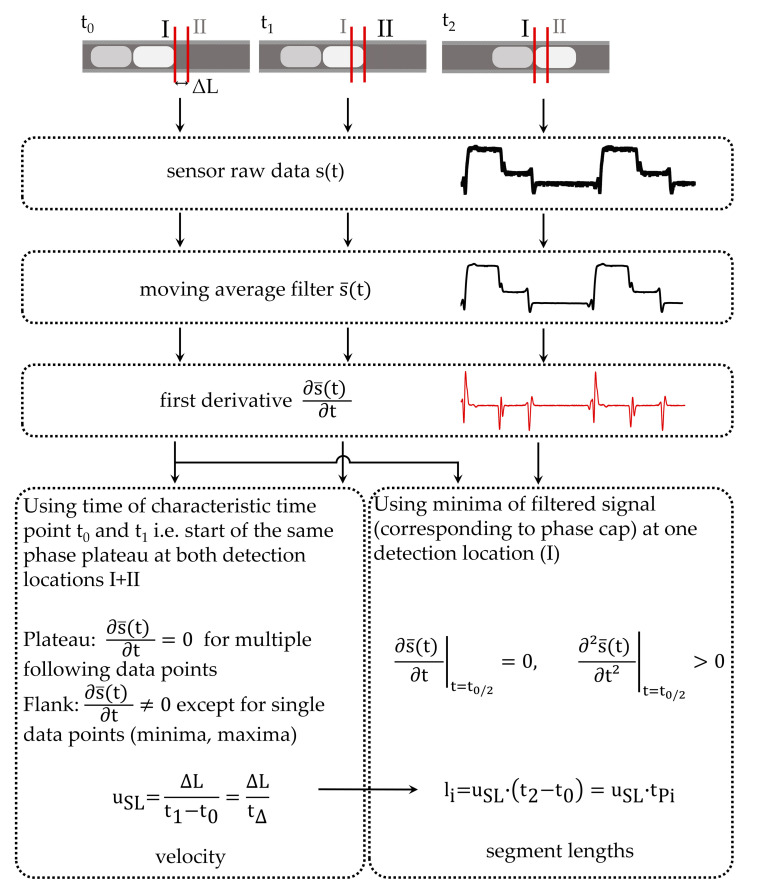
Mathematical approach to determine the velocity in a fully developed slug flow and the length of each phase segment. The velocity is determined by the characteristic time, and a new phase plateau can be seen to begin using the first derivative of the filtered signal. For calculating all segment lengths, the minimum points surrounding the corresponding phase plateau are utilized.

In Figure 12, the mathematic approach to determine the velocity and phase segment length is summarized.

### 3.3. Distance between Transmissive Sensors

An important factor influencing the measuring accuracy of the method presented is the distance between the photoelectric sensors. If this distance is too short, even the smallest fluctuations in the flow velocity can cause a large measurement error. If the distance is too large, one must ensure that the segments can be tracked correctly, since several bubbles and slugs may be located between both sensors. For this reason, several distances between the transmissive sensors were chosen and the resulting velocities were compared with camera recordings. The smallest distance is 0.8 mm, which is determined by the dimensions of the double transmissive sensor unit (TCUT1300X01, Vishay). With a second transmissive sensor, greater gaps can be realized. When the spacing between the transmissive sensors is larger than a phase triplet, a third transmissive sensor is used. This is placed at the minimum distance (0.8 mm) to the first transmissive sensor, so that an approximate velocity of the flow can be captured. With the distance to the third transmissive sensor, the time can be estimated when the slug or bubble observed flows into the third transmissive sensor; hence, a phase segment can be followed over several millimeters. Thus, spacings between the transmissive sensors of 0.8–23 mm were compared. The methods described in Section 3.2 were used to determine the velocity.

**Figure 13:**
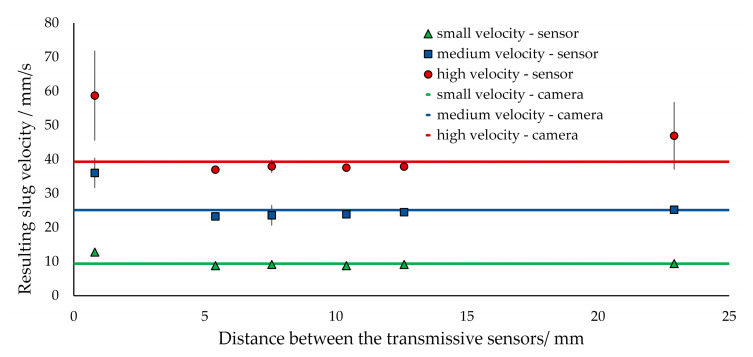
Comparison of measured velocities in a biphasic segmented slug flow (water, 1-hexanol) with respect to different spacings between the two essential transmissive sensors. The velocities are compared with data from the high-speed camera together with its standard derivations.

Figure 13 illustrates the measured velocities at different volumetric flow rates and transmissive sensor separations. Beginning with the smallest transmissive sensor distance of 0.8 mm, a deviation from the actual value can be seen, on the one hand, while, on the other hand, they also exhibit a large standard deviation. With low measuring frequencies, this could be attributed to the inaccuracy in the time parameter. However, with the measurement frequency of 9.6 kHz used here, this error can be assumed to be small. Slight fluctuations in the flow velocity lead to the deviations. These are caused, for example, by slug generation or by fluctuations in the gas volumetric flow from the mass flow controller used. If other liquid dosing systems, such as micro-gear pumps or peristaltic pumps, are utilized, these can create additional disturbances.

The transmissive sensor distances between 5 and 12.5 mm show good agreement with the actual flow velocities at all overall volumetric flow rates. For the largest spacing of 23 mm at high volumetric flow rates, a large deviation can also be detected. This is due to the fact that the estimation of the velocity via the first two transmissive sensors with a small gap between them is not sufficiently precise to track the slug or bubble observed up to the third transmissive sensor.

The accuracy of the photoelectric sensor separation is essential for a precise velocity determination. For example, if the distance used for calculation has an error of 5%, the velocity, according to Equation (1), deviates from its actual value by 5%. For a spacing of 5 mm, 5% corresponds to a deviation of 250 µm, a distance that is extremely difficult to measure. To minimize this error, an independent calibration of the distance is recommended. The difference between the measured velocities (transmissive sensor) and the actual ones (camera) can be minimized by varying the distance used in the calculation. The distance so determined has to be established only once for each pair of transmissive sensors. In order to define the distance in a consistent manner, it is recommended to solder both transmissive sensors on a single circuit board.

### 3.4. Experimental Validation

Since the accuracy of the measurement method plays a major role in the effective implementation of a feedback control, the measures devised are used in combination. The capillary is fixed at such a height within the capillary holder that all three phases can be easily distinguished (525 µm). The data evaluation via the time derivative of the measuring signal allows a robust determination of velocity and length. The distance between the transmissive sensors was chosen to be 6.5 mm. It is narrow enough to easily identify the phase segment and wide enough to decrease the deviation at very small distances. Under these conditions, the measured velocities and lengths from the transmissive sensor technique are compared with those of the camera images.

**Figure 14:**
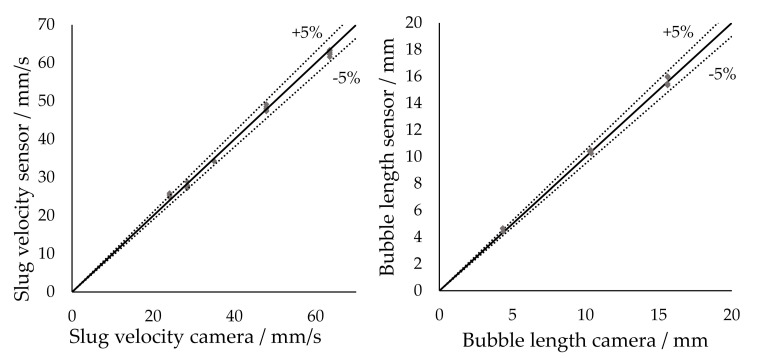
Parity diagrams to compare the performance of the transmissive sensor with the evaluation method presented to camera-determined values. Left: Slug velocity. Right: Bubble length.

Figure 14 shows parity diagrams for velocities and lengths. The measured values deviate from the actual values by a maximum of 5%, which is in the same range as the C^4^D sensor systems; however, the new method is additionally applicable to arbitrary triphasic and nonconductive segmented slug-flows.

## 4. Discussion

The optoelectric sensor together with the evaluation presented is a convincing alternative to the traditional 2D measuring methods, which have to handle a much higher amount of data. Due to a flexible capillary positioning, even a triphasic flow can be monitored with high accuracy. By combining the optoelectronic sensor with data evaluation on a microcontroller board, the characteristic values of the slug flow, i.e., the lengths and velocities of the slugs and bubbles, can be determined reliably and quickly with a satisfactory precision (<5% error). This noninvasive sensor can, therefore, be easily integrated into a feedback control. To increase the sensitivity of the sensor and to create an even bigger difference between the phase plateaus, a more sensitive detector could be utilized allowing voltages above 5 V. This would lead to a continuous symmetric function with a maximum for each phase at the centered capillary position. Nevertheless, the most sensitive position is probably not in a centered position considering three-dimensional refraction a light at the bubble caps.

The transmissive sensor may encounter limitations in some applications, for instance, when reactions or extractions exhibit rapid dynamics or considerable variations in refractive indices, when phases possess very close or identical optical properties, and for systems which absorb light strongly.

The evaluation method utilizing the first derivative of the sensor signals can cope with most of the variations that will be encountered in the refractive indices, since the phase plateau height is not taken into account in the evaluation process. For a considerable change in the refractive index, where the signal plateaus can no longer be unambiguously assigned to a given phase by means of the slope direction, the technique becomes unsuitable. In such cases, other independent methods to identify the individual phases are required. For example, the gas phase with its usually very low refractive index leading to constant plateau height or two local minima in the vicinity of the signal indicating two successive dispersed bubbles could be used for orientation purposes in interpreting the multiphase structure.

If the refractive indices of the participating phases are either very close or identical, so that no distinction can be drawn between them on the basis of the absorption characteristics, the automated discrimination between the phases becomes challenging. The absorption behavior of one phase can of course still be modified with the assistance of an inert dye. For an appropriate choice of the light source and, thus, its wavelength, one can then once again differentiate between the phases. In this instance, the characteristic minima in the signal form may no longer be observed.

In addition, systems showing strong absorption and little transmission may present problems. However, as long as one of the dispersed phases is the strongly absorbing medium, the phases can be attributed correctly. On the other hand, if the continuous phase is the culprit, light would no longer pass through the capillary. Such difficulties may be surmounted by employing a higher-intensity light source. Similar constraints also apply to flow measurements via image analysis with 2D cameras.

In this work, the wavelength was chosen such that the substances used absorb only small parts of the light and, thus, the signal form is dictated predominantly by the refraction of light and the geometry of the capillary and the bubbles. However, the wavelength can also be selected in such a way that one or more phases absorb the light and, thus, an even stronger differentiation of the phases becomes possible. This can be helpful if the refractive indices of the individual phases are very close to one another. Hence, an absorption of light in a certain component does not exclude this sensor. The absorption of a certain wavelength can also be used to evaluate the concentrations of individual components. For example, noninvasive residence time measurements or local conversions can be determined. In this case, the absorption and not the refraction is to be emphasized, such that an appropriate light source or a band filter must be used to guarantee the sensitivity sought. Via the combination of refraction, reflection, absorption, and the choice of the light source wavelength, this sensor can be applied to a huge number of possible substance systems.

In terms of cost, the optoelectric sensor is very cheap with a price of <1 USD. The use of microcontroller boards allows the simultaneous evaluation of several sensors with a single board or, as another option, one can integrate them directly into a control system. In a numbering up, new channels can be added flexibly to this system as required. A hierarchical control system, for example, is ideal for this purpose, in which the reactor conditions can be defined at a central location, but the control and sensor systems are implemented locally for individual capillaries or capillary bundles. An outsourcing of these sensor evaluations has the advantage that the sensors do not significantly occupy the computing power of the main control unit (e.g., a personal computer).

The sensor devised can be used in the future for various control tasks, for example, the control of slug or bubble lengths and volume flow rates already mentioned. If the actuators for such systems can also be implemented at very low cost, an industrial application of parallelized capillary reactors becomes a realistic, high-performance design strategy.

## Figures and Tables

**Table 1:** Refractive indices of the substances considered, with air being considered to have the same refractive index as nitrogen.

Substance	State of Aggregation	Refractive Index
Nitrogen/air [30]	Gaseous	1.003
Water [30]	Liquid	1.333
1-Hexanol [30]	Liquid	1.416
Fluorinated ethylene propylene (FEP) [31]	Solid	1.344

**Figure A1:**
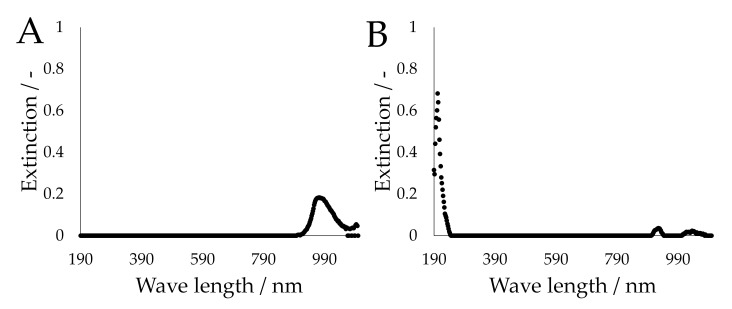
Ultraviolet–visible light (UV–Vis) spectra of water (**A**) and 1-hexanol (**B**). Reference medium: air.

## Appendix A

UV–Vis spectra (Figure A1) for the liquid phases 1-hexanol and water were recorded in order to neglect additional effects of absorption on the recorded signals (Genesys 10 UV, Thermo Electron, Waltham, MA, USA). At the wavelength of 940 nm used, only a small amount of light is absorbed; thus, the influence of absorption can be considered negligible.
